# Editorial: Compositional data analysis and related methods applied to genomics—a first special issue from *NAR Genomics and Bioinformatics*

**DOI:** 10.1093/nargab/lqaa103

**Published:** 2020-12-09

**Authors:** Ionas Erb, Gregory B Gloor, Thomas P Quinn

**Affiliations:** Centre for Genomic Regulation (CRG), The Barcelona Institute of Science and Technology, Dr Aiguader 88, 08003 Barcelona, Spain; Department of Biochemistry, Schulich School of Medicine & Dentistry, Western University, London, ON N6A 5C1, Canada; Applied Artificial Intelligence Institute, Deakin University, Waurn Ponds, VIC 3216, Australia

## THE ROCKY ORIGINS

It is our great pleasure to present this collection of articles on compositional data analysis (CoDA) to the readers of *NAR Genomics and Bioinformatics* (NARGAB). CoDA emerged as a discipline in the 1980s when John Aitchison laid out the basis of a statistical theory dealing with certain kinds of *constrained* data represented by proportions. Motivated by the geosciences, where data in the form of mass percentages often occur (e.g. in rock samples), Aitchison proposed a theoretical framework based on *ratios*, or rather—for reasons of symmetry—*log ratios* (1). Log-ratio analysis offered a solution to phenomena such as the negative bias in correlations that had puzzled researchers for at least a century. It has led to important developments in both practical analysis and underlying theory ever since.

Although developed within the geosciences, the latest application of CoDA is within the biosciences: the growing importance of experiments that aim to quantify the presence of hundreds to thousands of molecules has put a new spotlight on CoDA. Sequencing experiments produce *relative count data*, and although their nature differs from simple percentages, the application of CoDA has already shown to be advantageous for the analysis of transcriptome and microbiome data. Recent applications include reference-aware analysis of microbial compositions (2), their dynamics (3) and phylogenetic scales (4), reference-aware analysis (5) and simulation (6) of RNA-seq data, PCR bias correction (7), association (8) and differential network analysis (9) as well as feature selection (10) and model fitting (11). All these techniques apply more generally to positive-valued signal data, implying they could also be used in fields like proteomics (12) and metabolomics (13). This is just a small and somewhat arbitrary selection of some recent applications; for further reference, we refer the reader to reviews like (14,15,16).

Here, we aim to add to this growing corpus by inviting applications to genomics that make use of CoDA methods, either directly or indirectly. The purpose of this special issue is thus 2-fold: to showcase the utility of CoDA in genomics and to popularize the techniques among researchers who are not yet aware of them.

To motivate the compositional approach from a genomics perspective, let us discuss here a simplified example. Consider an experiment where we count the abundances of molecules belonging to a number of ‘species’ (representing, say, transcripts or bacteria). It is usually impossible to exhaustively count *all* molecules in an environment, having the (typically unknown) total *N*_1_. If our sample is unbiased and large enough, however, each species’ count reflects the relative proportion of molecules present in the environment. Thus, up to a factor of proportionality *N*_1_/*n*_1_ (where *n*_1_ is the total number of molecules counted), we have obtained a useful representation of our environment. While this might be the end of the story for a single sample, problems can occur when we want to compare this sample with others. For this, we need a common scale.

## THE QUEST FOR A COMMON SCALE

When are two samples of relative count data comparable?

One answer to this question is ‘When they are normalized appropriately’.

However, there are different notions of *normalization* used to make species counts comparable between samples. Let us assume we have designed our second experiment such that *n*_2_, the number where we stop counting molecules, is identical to the one in the first experiment. Since *n*_2_ = *n*_1_, in a certain sense these data are already normalized. When we do not mind losing data, this type of normalization can also be achieved for the case *n*_2_ ≠ *n*_1_ by downsampling the data of the sample with a greater *n*_*i*_ (rarefaction). An alternative strategy can be applied on any samples by dividing each species count in sample *i* by the total *n*_*i*_ (or, equivalently, by calculating transcripts per million). This yields *proportions*, the central object of interest in CoDA. Proportions have much in common with the positive counts of our genomics example. [This becomes clear when representing compositions as equivalence classes; see (17).] Is a comparison of proportions between experiments valid?

Well, it depends.

For one, it depends on what we want to achieve with our comparison. Although it is possible to compare proportions directly (or their composite measures like alpha diversity), we often cannot obtain all of the results we want. For example, as Pearson first noted, the evaluation of correlations between two species is misleading for proportions (18). There are also methodological problems for even the simplest research questions, such as ‘How did the abundance of a species change between environments?’ To answer this question without additional information, the two samples would need to have a common scale, i.e. reflect the scale of their original environments. Simple proportions do not generally have a common scale in this sense because, although we have control over *n*_1_ and *n*_2_, we usually do not know how they relate to *N*_1_ and *N*_2_. As such, the (unknown) proportionality factors we referred to above, which would be needed to calculate the *true abundances* for each species, may not be the same for both experiments. Assuming *N*_1_/*N*_2_ = *n*_1_/*n*_2_ could lead an analysis astray and often did (19).

For comparisons that should not be done on proportions, another normalization technique, called *effective library size normalization*, is a popular choice (20). In differential gene expression analysis, this approach compares the (log) ratio of counts with respect to a *reference* species known to approximate the same number of molecules in both populations. These ratios are compared instead of the counts (or their proportions). If such a species is not known, the reference can be replaced by a suitably robust composite measure (i.e. a *pseudo-reference* species) obtained from various species assumed not to have changed *en masse*. One such measure is the geometric mean over all counts in the sample [see the supplement to (15)], and the assumption needed to put counts on a common scale can be stated as follows: A suitably defined aggregate of the species does not change *between their original environments*. This usually means that the majority of species exhibited only stochastic change.

Interestingly, effective library size normalization was developed independently of CoDA. Yet, it is analogous to a fundamental CoDA technique known as the log-ratio transformation (with the alr transformation using a single reference species and the clr transformation using the geometric mean of all species instead). While log-ratio transformations can be used to normalize data to an *effective library size*, they were not designed for it. Their purpose is first and foremost to remove the *constant-sum constraint* from the samples, thus enabling an unconstrained analysis on the real numbers. Put differently, whenever we consider proportions for a sample *i*, a fixed *n_i_* introduces dependencies between our variables, such that an increase in counts for one species requires a decrease in counts for all other species (i.e. so that *n_i_* remains constant). Log-ratio transformations remove this constraint. When combined with the normalization assumptions mentioned above, they can also be understood as putting data on a common scale.

Without these assumptions, log-ratio transformations still lead to valid statistical analyses; however, the results are somewhat harder to interpret because the reference changes the nature of the original variables. But such ratios can also be used for an alternative strategy that does not need the notion of a reference. Note that comparing pairs of species between samples via their ratio entirely circumvents the questions of scale and reference. Sometimes species ratios have a direct meaning to the practitioner, or, in the case of genes, can be interpreted in terms of stoichiometric change.

## TO NEW FRONTIERS

Here, we provide a brief glimpse of the 10 papers contained in this special issue. One of the first steps in each data analysis should be a visualization to explore their most obvious characteristics. Two of the present articles deal with the issue of visualizing compositional data. Fedarko *et al.* (21) present a convenient interface called ‘Qurro’ for ranking features with respect to their differential abundance according to user-specified log ratios. Among other things, this allows the user to interactively explore how different features can serve as reference frames for relative data analysis. Hawinkel *et al.* (22) present ‘COMBI’, an R package that enables visualizations of multi-omics datasets making use of multi-plots obtained from latent variable models. Such multi-plots are a generalization of the well-known biplots that are popular in CoDA. They are an effective way to incorporate covariates in the visualization provided by the latent variables.

As mentioned earlier, the data obtained from sequencing experiments are not compositional in a strict sense; rather, they are counts whose size contains information about measurement accuracy. There are two articles that explore the implications of this. Egozcue *et al.* (23) revisit the distributional modeling of count compositions. While providing a short review of current approaches, they also make a proposal for a new class of distributions with interesting properties. An emblematic application is discussed: PCR bias as a bottleneck problem in sequencing library preparation. Lovell *et al.* (24) show that compositional measures of association, like proportionality, run into problems when ignoring the discrete nature of the data, especially for small counts where count size gains greater importance. Measures of proportionality can fluctuate considerably here, and the deviations from what is obtained using continuous compositions (where exact proportional relationships are possible) can no longer be ignored. Meanwhile, Badri *et al.* (25) likewise explore proportionality and other measures of compositional association. They show how shrinkage estimation, a statistical regularization technique, can improve the detection of true taxon–taxon associations for sparse microbiome count data. Taken together, these studies further our understanding of how to model count data using compositional techniques. Such count data differ from the continuous compositional data that dominated the geosciences.

One of the most important problems when analyzing single-cell RNA-seq data is the correct inference of cell types. Wu *et al.* (26) present a new promising clustering algorithm that exploits the merits of the *L*–∞ distance on clr-transformed RNA-seq data.

Although the clr transformation allows for an unconstrained analysis, its application in the absence of a normalizing assumption can challenge interpretability. Thus, there exists a strong motivation to find alternatives to the clr. Lin *et al.* (27) propose one such alternative, an algorithm that seeks to identify genes that are stably expressed in single-cell RNA-seq data, and to use them as an internal reference to normalize the data. Two more articles discuss *normalization-free* alternatives to the clr that aim to learn interpretable log ratios directly from the data. Susin *et al.* (28) show how the ‘selbal’ package can learn a single parsimonious log contrast of species, called a *balance*, that differentiates samples. Quinn and Erb (29) introduce a package called ‘amalgam’ that sums species in a data-driven way to construct *summed log ratios* that likewise differentiate samples. Since neither method relies on a clr, either could provide an alternative to differential expression analysis in the case that the majority of genes *do* change.

Last but not least, Sisk-Hackworth and Kelley (30) present a complete CoDA re-analysis of a multi-omics time-series dataset. By examining associations within bacterial communities, as well as associations between bacteria and metabolites via multi-omics integration, their study provides a clear example of how existing clr and non-clr methods can be adopted for real-world applications.

This special issue is designed in form of an open article collection. This means that it is only the beginning of an ongoing series within the NARGAB universe, where new CoDA-related research can be contributed at any time. We hope to have met the interest of our readers with this selection, and look forward to their future contributions to this dynamic and widely open field.

## References

[1] Aitchison J. The Statistical Analysis of Compositional Data. 1986; London Chapman & Hall.

[2] Morton J.T., Marotz C., Washburne A., Silverman J., Zaramela L.S., Edlund A., Zengler K., Knight R. Establishing microbial composition measurement standards with reference frames. Nat. Commun. 2019; 10:2719. 31222023 10.1038/s41467-019-10656-5 PMC6586903

[3] Martino C., Shenhav L., Marotz C.A., Armstrong G., McDonald D., Vázquez-Baeza Y., Morton J.T., Jiang L., Dominguez-Bello M.G., Swafford A.D. et al. Context-aware dimensionality reduction deconvolutes gut microbial community dynamics. Nat. Biotechnol. 2020; doi:10.1038/s41587-020-0660-7. 10.1038/s41587-020-0660-7 PMC7878194 32868914

[4] Silverman J.D., Washburne A.D., Mukherjee S., David L.A. A phylogenetic transform enhances analysis of compositional microbiota data. eLife. 2017; 6:e21887. 28198697 10.7554/eLife.21887 PMC5328592

[5] Quinn T.P., Erb I., Gloor G., Notredame C., Richardson M.F., Crowley T.M. A field guide for the compositional analysis of any-omics data. GigaScience. 2019; 8:giz107. 31544212 10.1093/gigascience/giz107 PMC6755255

[6] McGee W.A., Pimentel H., Pachter L., Wu J.Y. Compositional data analysis is necessary for simulating and analyzing RNA-seq data. 2019; 02 March 2019, preprint: not peer reviewed 10.1101/564955.

[7] McLaren M.R., Willis A.D., Callahan B.J. Consistent and correctable bias in metagenomic sequencing experiments. eLife. 2019; 8:e46923. 31502536 10.7554/eLife.46923 PMC6739870

[8] Skinnider M.A., Squair J.W., Foster L.J. Evaluating measures of association for single-cell transcriptomic*s*. Nat. Methods. 2019; 16:381–386. 30962620 10.1038/s41592-019-0372-4

[9] McGregor K., Labbe A., Greenwood C.M.T. MDiNE: a model to estimate differential co-occurrence networks in microbiome studies. Bioinformatics. 2020; 36:1840–1847. 31697315 10.1093/bioinformatics/btz824 PMC7075537

[10] Quinn T.P., Erb I. Interpretable log contrasts for the classification of health biomarkers: a new approach to balance selection. mSystems. 2020; 5:e00230-–19. 32265314 10.1128/mSystems.00230-19 PMC7141889

[11] Bates S., Tibshirani R. Log-ratio lasso: scalable, sparse estimation for log-ratio models. Biometrics. 2019; 75:613–624. 30387139 10.1111/biom.12995 PMC9470385

[12] O’Brien J.J., O’Connell J.D., Paulo J.A., Thakurta S., Rose C.M., Weekes M.P., Huttlin E.L., Gygi S.P. Compositional proteomics: effects of spatial constraints on protein quantification utilizing isobaric tags. J. Proteome Res. 2018; 17:590–599. 29195270 10.1021/acs.jproteome.7b00699 PMC5806995

[13] Kalivodová A., Hron K., Filzmoser P., Najdekr L., Janečková H., Adam T. PLS‐DA for compositional data with application to metabolomics. J. Chemom. 2015; 29:21–28.

[14] Gloor G.B., Macklaim J.M., Pawlowsky-Glahn V., Egozcue J.J. Microbiome datasets are compositional: and this is not optional. Front. Microbiol. 2017; 8:2224. 29187837 10.3389/fmicb.2017.02224 PMC5695134

[15] Quinn T.P., Erb I., Richardson M.F., Crowley T.M. Understanding sequencing data as compositions: an outlook and review. Bioinformatics. 2018; 34:2870–2878. 29608657 10.1093/bioinformatics/bty175 PMC6084572

[16] Greenacre M. Compositional data analysis. Annu. Rev. Stat. Appl. 2021; 8:1–27.

[17] Barceló-Vidal C., Martín-Fernández J.-A. The mathematics of compositional analysis. Austrian J. Stat. 2016; 45:57–71.

[18] Pearson K. Mathematical contributions to the theory of evolution—on a form of spurious correlation which may arise when indices are used in the measurement of organs. Proc. R. Soc. Lond. 1897; 60:489–498.

[19] Lovén J., Orlando D.A., Sigova A.A., Lin C.Y., Rahl P.B., Burge C.B., Levens D.L., Lee T.I., Young R.A. Revisiting global gene expression analysis. Cell. 2012; 151:476–482. 23101621 10.1016/j.cell.2012.10.012 PMC3505597

[20] Robinson M.D., Oshlack A. A scaling normalization method for differential expression analysis of RNA-seq data. Genome Biol. 2010; 11:R25. 20196867 10.1186/gb-2010-11-3-r25 PMC2864565

[21] Fedarko M.W., Martino C., Morton J.T., González A., Rahman G., Marotz C.A., Minich J.J., Allen E.E., Knight R. Visualizing ’omic feature rankings and log-ratios using Qurro. NAR Genomics Bioinformatics. 2020; 2:lqaa023. 32391521 10.1093/nargab/lqaa023 PMC7194218

[22] Hawinkel S., Bijnens L., Cao K.-A.L., Thas O. Model-based joint visualization of multiple compositional omics datasets. NAR Genomics Bioinformatics. 2020; 2:lqaa050. 10.1093/nargab/lqaa050 PMC7671331 33575602

[23] Egozcue J.J., Graffelman J., Ortego M.I., Pawlowsky-Glahn V. Some thoughts on counts in sequencing studies. NAR Genomics Bioinformatics. 2020; 2:lqaa094. 10.1093/nargab/lqaa094 PMC7679068 33575638

[24] Lovell D.R., Chua X.-Y., McGrath A. Counts: an outstanding challenge for log-ratio analysis of compositional data in the molecular biosciences. NAR Genomics Bioinformatics. 2020; 2:lqaa040. 10.1093/nargab/lqaa040 PMC7671413 33575593

[25] Badri M., Kurtz Z.D., Bonneau R., Müller C.L. Shrinkage improves estimation of microbial associations under different normalization methods. NAR Genomics Bioinformatics. 2020; doi:10.1093/nargab/lqaa100. 10.1093/nargab/lqaa100 PMC7745771 33575644

[26] Wu H., Mao D., Zhang Y., Chi Z., Stitzel M., Ouyang Z. A new graph-based clustering method with application to single-cell RNA-seq data from human pancreatic islets. NAR Genomics Bioinformatics. 2020; doi:10.1093/nargab/lqaa087. 10.1093/nargab/lqaa087 PMC7803008 33575647

[27] Lin L., Song M., Jiang Y., Zhao X., Wang H., Zhang L. Normalizing single-cell RNA sequencing data with internal spike-in-like genes. NAR Genomics Bioinformatics. 2020; 2:lqaa059. 10.1093/nargab/lqaa059 PMC7671304 33575610

[28] Susin A., Wang Y., Cao K.-A.L., Calle M.L. Variable selection in microbiome compositional data analysis. NAR Genomics Bioinformatics. 2020; 2:lqaa029. 10.1093/nargab/lqaa029 PMC7671404 33575585

[29] Quinn T.P., Erb I. Amalgams: data-driven amalgamation for the dimensionality reduction of compositional data. NAR Genomics Bioinformatics. 2020; 2:lqaa076. 10.1093/nargab/lqaa076 PMC7671324 33575624

[30] Sisk-Hackworth L., Kelley S.T. An application of compositional data analysis to multiomic time-series data. NAR Genomics Bioinformatics. 2020; 2:lqaa079. 10.1093/nargab/lqaa079 PMC7671389 33575625

